# GDF15 AND REDUCED PHYSICAL FUNCTION FOLLOWING TOTAL KNEE REPLACEMENT: A STUDY OF PHYSICAL RESILIENCE AND AGING

**DOI:** 10.1093/geroni/igad104.3366

**Published:** 2023-12-21

**Authors:** William Fountain, Nicholas Milcik, Nicholas Schmedding, Frederick Sieber, Julius Oni, Ravi Varadhan, Karen Bandeen-Roche, Jeremy Walston

**Affiliations:** Johns Hopkins University, Baltimore, Maryland, United States; Johns Hopkins University, Baltimore, Maryland, United States; Johns Hopkins University, Baltimore, Maryland, United States; Johns Hopkins University, Baltimore, Maryland, United States; Johns Hopkins University, Baltimore, Maryland, United States; Johns Hopkins University School of Medicine, Baltimore, Maryland, United States; Johns Hopkins University, Baltimore, Maryland, United States; Johns Hopkins University, Baltimore, Maryland, United States

## Abstract

The metabolic and inflammatory cytokine growth-differentiation factor 15 (GDF15) increases with age and negatively associates with physical and cognitive function in older adults. We hypothesized GDF15 also negatively associates with resilient outcomes after surgery. This hypothesis was tested in the SPRING study of physical resiliency after total knee replacement by assessing relationships between pre-operative plasma GDF15 levels and postoperative resilience measures including short physical performance battery (SPPB) scores, fatigability, and grip strength. GDF15 analyses and physical resilience were assessed in 127 SPRING participants (age 70±6 yrs, n=83 women). Baseline GDF15 levels correlated with age (r=0.263, P< 0.05); age-adjusted analyses were applied. In total, pre-operative GDF15 levels did not significantly correlate with functional measures at any timepoint. However, in men (n=44), GDF15 levels correlated with age (r=0.408, P< 0.05) and significant age-adjusted correlations were observed in fatigue (r=0.368, P< 0.05), gait speed (r=-0.428, P< 0.05), and SPPB score (r=-0.329, P< 0.05). Additionally, GDF15 levels correlated with chair stands at six months (r=-0.398, P< 0.05), SPPB score at six months (r=-0.384, P< 0.05), and gait speed after one year (r=-0.487, P< 0.05). GDF15 did not significantly correlate with changes in SPPB score (P>0.05). There were no significant correlations between GDF15 and any of these functional measures in women (r-value range: -0.259 to 0.107; p-value range: 0.112 to 0.969). Elevated pre-operative GDF15 appears to correlate with worsening physical function following knee replacement in men, but not women. Further investigation is necessary to understand the relationship between GDF15 and the biology of physical resiliency.

